# Quantifying workload using nonlinear dynamical measures of biomechanical parameters during cycling on a roller trainer

**DOI:** 10.1371/journal.pone.0285408

**Published:** 2023-05-09

**Authors:** Ann-Kathrin Harsch, Alexander Kunert, Daniel Koska, Christian Maiwald

**Affiliations:** 1 Institute of Human Movement Science and Health, Department of Research Methodology and Data Analysis in Biomechanics, Chemnitz Universitiy of Technology, Chemnitz, Germany; 2 Institute for Mechanical and Plant Engineering ICM, Chemnitz, Germany; Federal University of Technology - Parana, BRAZIL

## Abstract

The aim of the present study was to determine the effectiveness of nonlinear parameters in distinguishing individual workload in cycling by using bike-integrated sensor data. The investigation focused on two nonlinear parameters: The *ML*1, which analyzes the geometric median in phase space, and the maximum Lyapunov exponent as nonlinear measure of local system stability. We investigated two hypothesis: **1.** *ML*1_*α*_, derived from kinematic crank data, is as good as *ML*1_*F*_, derived from force crank data, at distinguishing between individual load levels. **2.** Increasing load during cycling leads to decreasing local system stability evidenced by linearly increasing maximal Lyapunov exponents generated from kinematic data.

A maximal incremental cycling step test was conducted on an ergometer, generating complete datasets from 10 participants in a laboratory setting. Pedaling torque and kinematic data of the crank were recorded. *ML*1_*F*_, *ML*1_*α*_, and Lyapunov parameters (λ_*st*_, λ_*lt*_, *ι*_*st*_, *ι*_*lt*_) were calculated for each participant at comparable load levels. The results showed a significant linear increase in *ML*1_*α*_ across three individual load levels, with a lower but still large effect compared to *ML*1_*F*_. The contrast analysis also confirmed a linearly increasing trend for λ_*st*_ across three load levels, but this was not confirmed for λ_*lt*_. However, the intercepts *ι*_*st*_ and *ι*_*lt*_ of the short- and longterm divergence showed a statistically significant linear increase across the load levels.

In summary, nonlinear parameters seem fundamentally suitable to distinguish individual load levels in cycling. It is concluded that higher load during cycling is associated with decreasing local system stability. These findings may aid in developing improved e-bike propulsion algorithms. Further research is needed to determine the impact of factors occurring in field application.

## Introduction

When using e-bikes, the control and regulation of propulsion is important for ensuring a natural riding experience and for optimizing range. However, users can often only specify a level of support relative to their own pedaling power by selecting a predetermined level. Thus, the user’s individual effort is not factored into the algorithms generating output levels. To develop more sophisticated algorithms based on individual effort, parameters are needed that allow the adequate characterization of individual load properties. Preferably, these should be based on information from sensors that are mechanically integrated into the bike, rather than additional physiological devices like heart rate monitors.

Biomechanical studies in cycling often analyze pedaling movement behavior to optimize force transmission or training techniques using linear and nonlinear methods. Nonlinear methods are applied to examine the relationship between external load and movement behavior by analyzing changes in cadence, pedal torque on incline, or higher power output [1–5]. Nonlinear dynamic methods can offer an added value in this context, since they consider the spatio-temporal evolution of a system. Compared to linear methods, fluctuations do not necessarily represent noise, but may indicate underlying properties of the investigated system [6].

A basis for the application of nonlinear methods in pedaling was provided by Quintana-Duque and Saupe [7], who showed that knee motion data in indoor pedaling underlies deterministic chaos and that a chaotic dynamical system can be assumed. More advanced nonlinear methods, for example, can distinguish different body positions via time variability (changing behavior in cycle duration) [8]. Furthermore, non-physiological parameters, such as crank torque variability [9], seem to be effected by pedaling intensity.

In previous studies [10, 11], we analyzed crank forces and developed a nonlinear parameter that correlates with the individual load when, for example, changes in terrain inclination are prevalent. This parameter, called *ML*1, is defined by the vector length of the geometric median of the force points in two dimensional phase space. It is also associated with a physiological parameter (heart rate), but shows less delay in response to terrain-induced load changes. This was already demonstrated in laboratory and field tests. Therefore, *ML*1 appears to be suitable for being integrated into control systems of e-bike propulsion. However, attaching a force sensor to the crank is expensive. A more affordable alternative could be to integrate inertial sensors, such as gyroscopes, to the crank arm. Therefore, the first question of the present work is to investigate the extent to which the geometric median calculated via kinematic signals (*ML*1_*α*_) agrees with the geometric median from force data (*ML*1_*F*_) and can also distinguish individual load levels.

Variability, stability, and/or complexity of a movement are examined within the context of nonlinear dynamic system analysis in biomechanics [12]. These terms are related, but should not be interchanged. In the present paper, we follow van Emmerik et al. [6], from the field of gait analysis, and define the local stability of a movement system as the resistance to small perturbations. In gait analyses, local system stability is often quantified using the maximum Lyapunov exponent λ_*max*_ [13–15]. λ_*max*_ describes the rate of divergence of originally proximate points on trajectories in multidimensional phase space. Thus, λ_*max*_ characterizes the system’s sensitivity to local perturbations [16]. The question remains whether the Lyapunov approach can be used to quantify load-dependent changes in pedaling behavior when kinematic data from accelerometers on the crank are considered (research question II).

The overall aim of this paper is to determine the extent to which nonlinear methods in combination with kinematic data, are suited to describe individual load in cycling. In addition to the first research question, regarding the extent of agreement between *ML*1_*α*_ and *ML*1_*F*_ (1), we investigate how the local dynamic stability as quantified by the maximum Lyapunov exponent is able to distinguish between individual load levels (2).

## Materials and methods

### Participants

15 healthy participants were informed verbally and in writing about the content of the study and then gave written approval to participate. It was approved by the Ethics Committee at Chemnitz University of Technology (reference number V-337–17-CM-Radsport-17052019).

Previous studies have investigated heterogeneous groups [10, 11]. Therefore, we did not focus on typical e-bike users to generate a more homogenous group regarding age and ability. To ensure good endurance capacity of the participants, only active male cyclists and triathletes with at least 5 h/week training volume were included in the study. Training volume and anthropometric data for the cyclists in this study are shown in Table 1.

**Table 1:** Training volume and anthropometric data for the cyclists (all male).

Parameter	Mean	SD
*Age*[*years*]	37.1	7.0
*Height*[*cm*]	180.8	7.1
*Mass*[*kg*]	74.9	7.0
*Training*[*h*/*week*]	8.4	3.6

### Experimental setup

The participants performed a maximal incremental cycling test. Power output started at 100 W and was increased incrementally by 20 W each 3 minutes until the cyclists stopped the test due to exhaustion or permanently fell below a cadence of 60 cycles per minute. Therefore, each participant finished an individual number of intervals. No further restrictions regarding cadence (for example by using a metronome) were implemented, since Warlop et al. (2013) indicate that this can modify nonlinear properties of the pedaling behavior [12]. All tests were performed on the same bicycle (Stein Bikes Mauna Loa 29AL, Stein Bikes Zweiradhandel GmbH, Chemnitz) mounted on a roller trainer (Cyclus 2, RBM elektronik-automation GmbH). The power output was controlled using the Cyclus 2 software. All participants used the same non-cleat platform pedals to ensure comparable force transmission. Before starting the test, participants determined their individual saddle height. This could be adjusted after a 10 min warm up period, which was standardized to 100 W.

The raw pedaling torque was measured using a Stages Shimano XT Powermeter (Stages Inc., USA) with a crank length of 175 mm and a sampling rate of 64 Hz. Heart rate was recorded using a standard chest strap (Berry King, bestbeans UG, Germany). Raw data were transferred to a Windows PC using bluetooth protocol and were recorded in LabView (National Instruments, USA). Crank kinematics were captured using a gyroscope with an internal data logger (Envisible Dialogg Steinbeis-Forschungszentrum Human Centered Engineering, Chemnitz, DEU), which was attached to the left crank arm and recorded angular velocities at a sampling rate of 500 Hz. To control for fatigue, respiratory gases were analyzed with a spirometer (Oxycon Pro, Erich Jaeger, Germany). Exclusion criterion was a respiratory quotient greater than 1.

### Data analysis

All data analyses were performed in R (version 4.2), using R Studio [17]. The analyses included crank angular velocity and force data.

Ten complete datasets were generated. Three participants had to be excluded due to technical difficulties and missing recorded data, caused by battery failure on the crank or chest strap. Two other trials were excluded because of implausible gyroscope data, and because of an implausible increase of one participant’s heart rate to 230 beats/min.

Raw velocity data were first filtered using a fourth-order low-pass Butterworth filter with a cut-off frequency of 120 Hz. To facilitate the calculation of Lyapunov parameters, time normalization was performed for the entire time series of each of the 3 minute power intervals. This involved counting the number of cycles (revolutions of the crank) in the current interval, which are defined as *n*_*cyc*_, and normalizing the entire 3-minute time series to the number of cycles multiplied by 100. Thus, the number of points $n_{I_{p}}$ per power interval *I*_*p*_ was:

$$\begin{array}{c} n_{I_{p}}=n_{\text{cyc}}\cdot 100 \end{array}$$ (1)

Hence, information about the relative duration of one cycle was preserved.

The number of fulfilled power intervals was different for each participant, depending on the participant’s maximum achieved power. The *relative power interval* *I*_*p*,*rel*_ was determined to ensure comparability between individually experienced loads. *I*_*p*,*rel*_ includes the interval at 25%, 50% and 75% of the power difference of the start and the highest fully completed interval (*I*_*p*,*max*_). This power difference is defined as Δ*P* = *I*_*p*,*max*_ − *I*_*p*,*start*_. For example, the 75% relative power interval *I*_*p*,75_ is then calculated as:

$$\begin{array}{c} I_{p,75}=I_{p,start}+0.75\cdot \Delta P \end{array}$$ (2)

For percentages between two intervals, the next higher level was set as *I*_*p*,*rel*_.

#### Assumptions and testing for nonlinearity

One assumption of the nonlinear methods applied in this study is the stationarity [16]. To ensure stationarity of the angular velocity signal, an acceleration measure was defined using pointwise differentiation of the gyroscope’s angular velocity signal Δ*ω*_*i*_:

$$\begin{array}{c} \alpha_{i}=\Delta \omega_{i}=\omega_{i+1}-\omega_{i} \end{array}$$ (3)

Another prerequisite for the use of nonlinear analysis is that the data originates from a nonlinear process. Therefore, we first determined whether the data were subject to linear stochastic noise or nonlinearity by using the surrogate data method suggested in [16, 18]. This method was implemented in the R-package nonlinearTseries [19]. The function surrogateTest() was applied to the whole time series of the filtered and normalized differentiated angular velocity data for each participant.

Within this function, the null hypothesis is tested, that the data originates from a Gaussian linear stochastic process. Surrogate data are generated by using a phase randomization procedure and values of a discriminating statistic for the original (input) data and the surrogate data are compared. The whole time series of the filtered and normalized acceleration data for each participant was used as original (input) data for the test. Graphical results of the discriminating statistic and the original series can be found in the (see: S2 Fig). The null hypothesis was rejected for all participants, therefore the prerequisites for applying nonlinear methods are fulfilled for the tested data.

#### Nonlinear parameter ML1

**Fig 1:**
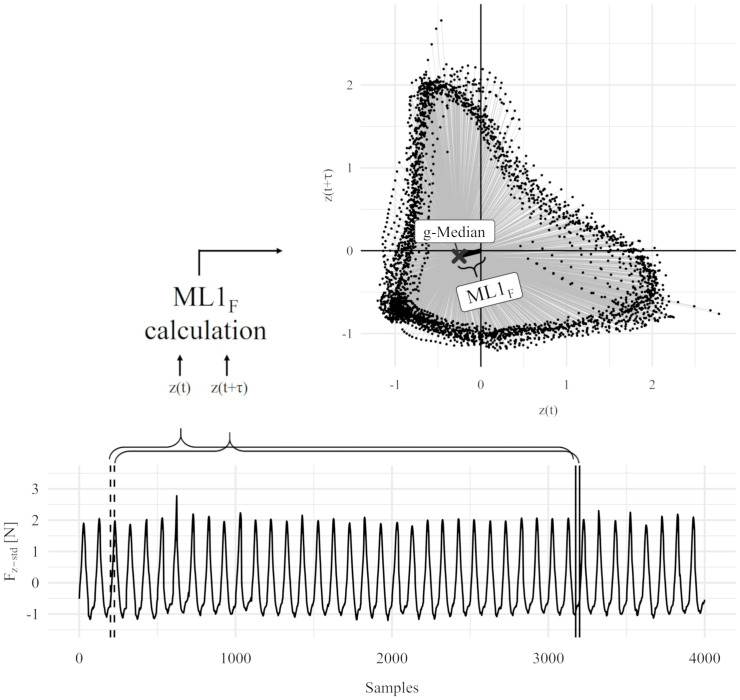
Method for calculating *ML*1 using the example *ML*1_*F*_. A sliding window considering 50 full z-standardized and time-normalized pedaling cycles to calculate *ML*1_*F*_ (see bottom of Fig 1; 30 instead of 50 pedaling-cycles are considered). The geometric median in 2-dimensional phase-space with a 25% delay was determined (see right side of Fig 1) for these cycles.

The novel *ML*1 method was applied to the pedaling forces using a sliding window algorithm [10, 11]. The window width was set to 50 normalized cycles (interpolation to 100 samples per cycle), representing 5000 points in phase space (see Fig 1). This resulted in a series of ML1 values in which each value represents the nonlinear characteristic of the last 50 force-time cycles. For each *I*_*p*,*rel*_, the mean of all *ML*1 values was calculated and used for further statistical analyses.

As input data for the *ML*1, the differentiated acceleration data (see Eq 3) of each *I*_*p*,*rel*_ was used to obtain the *ML*1_*α*_. To determine *ML*1_*F*_, force values were determined for the pedaling torque and time-normalized to 100 points per cycle. Furthermore, the force values were z-normalized across each *I*_*p*,*rel*_ of the incremental cycle test and the *ML*1 method was applied.

#### Nonlinear parameter Lyapunov-exponents

As mentioned earlier, λ_*max*_ characterizes the system’s sensitivity to local perturbations [16]. By calculating the divergence rate of points in phase space, the stability of the system is quantified. According to [20], the average divergence of neighboring points in phase space for discrete time steps can be described as:

$$\begin{array}{c} d_{j}\left(i\right)=C_{j}e^{\lambda_{max}\left(i\Delta t\right)} \end{array}$$ (4)

where *d*_*j*_(*i*) is the distance between the *j*th pair of nearest neighbors after *i* discrete time-steps, i.e., *i* ⋅ Δ*t* seconds, with Δ*t* denoting the sampling period of the time series. *C*_*j*_ describes the initial separation and λ_*max*_ depicts the growth rate.

In biomechanical analysis, experimental time series are investigated, thus the true maximum Lyapunov exponent cannot be determined—unlike fully describable systems of differential equations. λ_*max*_ is therefore defined as the slope of the linear range of the divergence curve. When taking the logarithm on both sides of Eq 4 and using a least-square fit to the average line over all values of *j*, denoted by 〈 〉, the largest Lyapunov exponent is defined by:

$$\begin{array}{c} \lambda_{max}=\frac{1}{\Delta t}\left\langle lnd_{j}\left(i\right)\right\rangle \end{array}$$ (5)

λ_*max*_ ≈0 corresponds to a stable system. If λ_*max*_ is positive, the system is divergent and thus unstable [16].

For experimental data, a distinction is made between the short-term and long-term divergence exponents. When observing the divergence curve in gait analysis, the short-term Lyapunov exponent λ_*st*_ represents the slope of the nearly linear range from the beginning up to the first step or stride. The long-term Lyapunov exponent λ_*lt*_ represents the gradient from the middle to final range (approximately 4th to 10th stride in gait). For the current analysis in cycling, we consider a stride analogous to a revolution of the crank as one cycle. The two Lyapunov measures should not be interpreted in the same way. According to Terrier and Reynard [21], in gait analysis, λ_*st*_ can be considered as a stability measure to prevent falls, while λ_*lt*_ is a measure of (gait) complexity.

**Fig 2:**
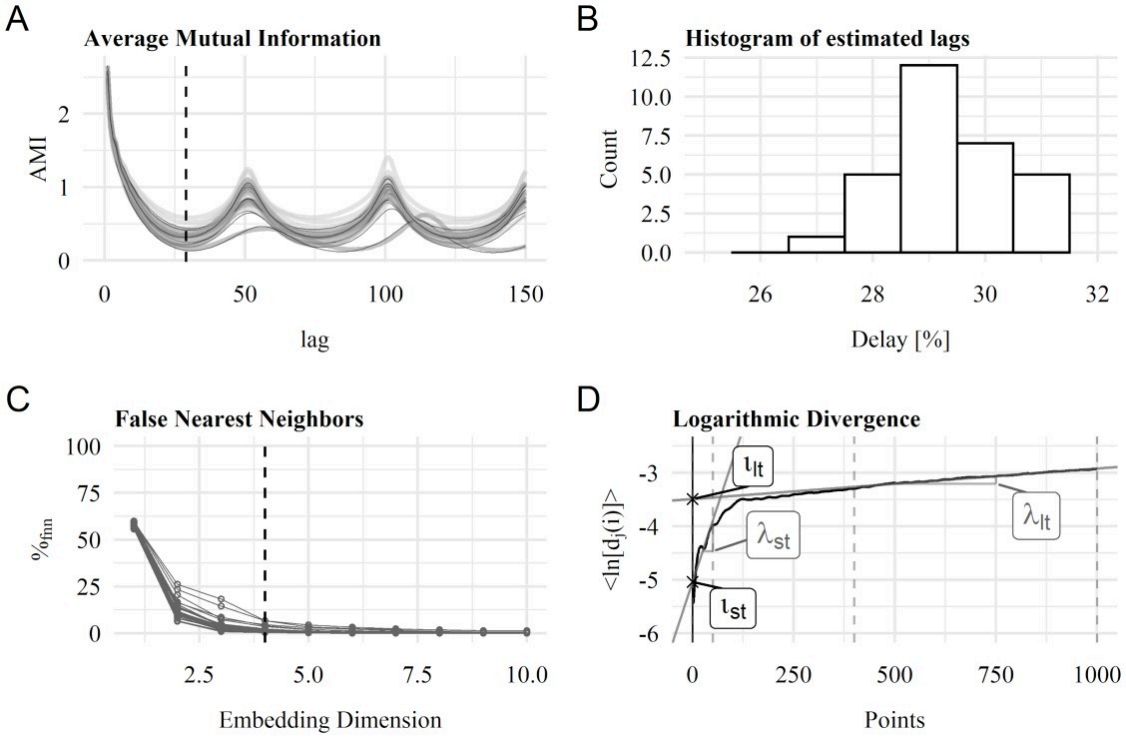
Method for calculating Lyapunov-exponent. **A:** Average Mutual Information (AMI) for all time series to determine overall delay. **B:** Distribution of considered delays as first AMI minimum. Overall delay was set to *τ* = 29. **C:** Calculation of False Nearest Neighbors to define overall embedding dimension, which was stated as *ed* = 4. **D:** Logarithmic divergence for calculating the short-term Lyapunov exponent as the slope for the first 100 points (λ_*st*_) and long-term Lyapunov exponent as slope for 400 to 1000 points (λ_*lt*_). Short-term and long-term intercepts were also considered.

During the present data analysis, λ_*st*_ and λ_*lt*_ were evaluated using the R-package tseriesChaos [22]. In a first step, state spaces with corresponding time delays *τ* were calculated using the Average Mutual Information (AMI) method. Compared to the autocorrelation function, the AMI also considers nonlinear correlations [23]. Different AMI lags running from 0 to 150 are shown in Fig 2A for all included time series. The first minimum was considered the appropriate time delay [16]. The distribution of all first minima is shown in the histogram in Fig 2B. For further calculation, *τ* = 29 was assumed for all participants and relative power intervals. To generate a suitable state space, an appropriate embedding dimension was needed in addition to *τ*. To find an optimal embedding dimension, we applied the False Nearest Neighbors (FNN) method. Figuratively, close points on an unfolded attractor can be far away from each other in the next higher dimension and are defined as False Nearest Neighbors [24]. The proportion of these FNN in one dimension is determined within the applied function. Close time separations or distances between two points are highly correlated and must be excluded when determining FNN. In simple terms, this is defined by the Theiler window, which was set to 3*τ [25]. The diameter of the neighborhood in which (False) Neighbors should be found was set to half the standard deviation of the time series [23]. Based on these FNN calculations (see Fig 2C), an embedding dimension of 4 was specified for all participants and intervals.

Finally, the divergence and the regression coefficients for λ_*st*_ and λ_*lt*_ were calculated with the functions lyap_k() and lyap(). Similar to gait analysis [13–15, 26–28], λ_*st*_ was determined as the slope of the exponential divergence for half a cycle (approximately 50 points). λ_*lt*_ was calculated as the slope of the divergence for cycles 4–10 (approximately points 400–1000). This method is shown in Fig 2D. The regression coefficients of the short-term and long-term divergence also include the intercepts *ι*_*st*_ and *ι*_*lt*_. Lyapunov parameters were calculated considering the whole 3-min time series of the *I*_*p*,*rel*_.

To obtain a common measure of the time series’ dispersion, the standard deviation *SD* was determined for each *I*_*p*,*rel*_.

### Statistical analysis

The calculated parameters (*ML*1_*F*_, *ML*1_*α*_, λ_*st*_, λ_*lt*_,*ι*_*st*_, *ι*_*lt*_, *SD*) were analyzed descriptively and tested for linear increase across the three *I*_*p*,*rel*_ using a contrast analysis. The contrast analysis is a special case of the analysis of variance. The alternative hypothesis is formulated via contrasts (i.e. lambda weights). A linear increase over the three individual relative power intervals *I*_*p*,*rel*_ was assumed for all determined parameters. Consequently, lambda weights of λ = {−1, 0, 1} were chosen. Contrast analyses of the parameters were performed using the calc_contrast()-function of the package cofad [29] for within-subject designs. Assumptions of normal distribution and homoscedasticity were assessed using boxplots. Symmetric boxes were interpreted as normally distributed data and interquartile ranges of equal length were interpreted as confirmation of variance homogeneity between groups.

## Results

### Incremental cycling test

Results of the incremental cycling test are shown in Table 2, reporting power outputs (in W per kg bodyweight), including means and standard deviations across the *I*_*p*,*rel*_. The absolute mean values of the power outputs increased consistently over the relative power intervals and the standard deviations remained approximately constant. The corresponding table including raw power and the figure with individual depicted development across the *I*_*p*,*rel*_ can be found in the supporting information (see: S1 Fig and S1 Table).

**Table 2:** Power outputs across relative power intervals.

P	*I*_*p*,25_[*W*/*kg*]	*I*_*p*,50_[*W*/*kg*]	*I*_*p*,75_[*W*/*kg*]
*P*01	2.473	3.091	3.709
*P*02	2.198	2.747	3.571
*P*03	2.169	2.892	3.614
*P*04	2.184	2.730	3.549
*P*05	2.011	2.682	3.575
*P*06	2.192	2.740	3.288
*P*07	2.307	2.884	3.461
*P*08	1.975	2.469	3.210
*P*09	2.483	3.310	4.138
*P*10	2.302	2.878	3.741
*mean*	2.229	2.842	3.586
*SD*	0.160	0.220	0.243

**Fig 3:**
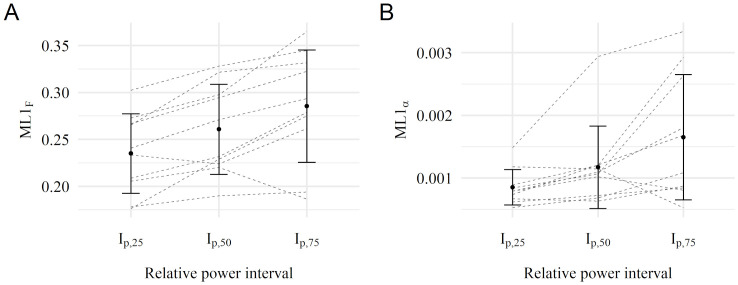
Results of the ML1 calculation. Individual participants are displayed as dashed lines. **A:** *ML*1_*F*_ across relative power intervals using crank force data. **B:** *ML*1_*α*_ across relative power intervals using crank acceleration data.

**Fig 4:**
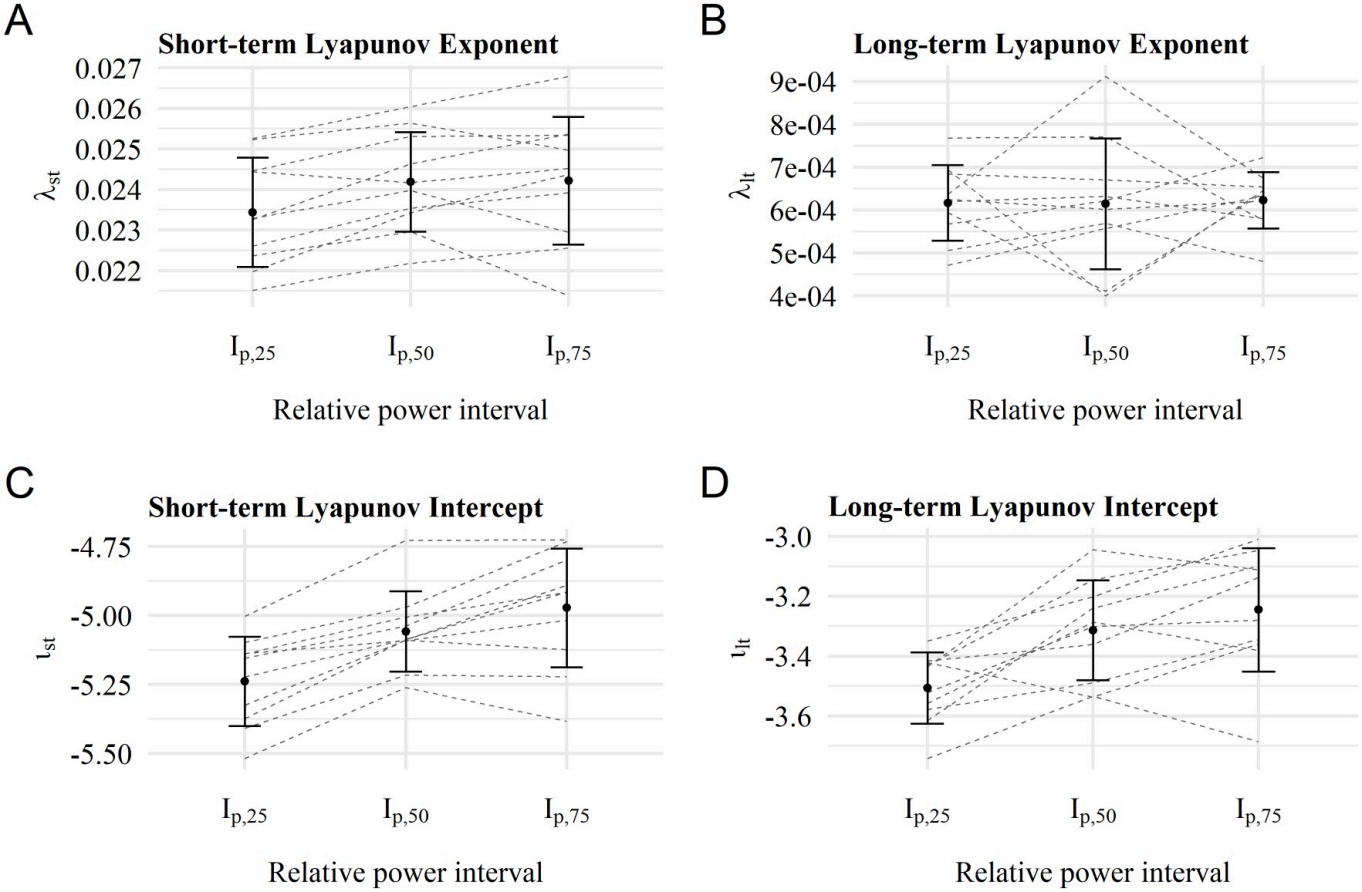
Results of the Lyapunov calculation. **A:** Short-term Lyapunov exponents as slope λ_*st*_ across relative power intervals show a slight increasing trend. **B:** Long-term Lyapunov exponents as slope λ_*lt*_ across relative power intervals show no trend. **C:** Short-term Lyapunov intercepts *ι*_*st*_ show an increasing trend across relative power intervals. **D:** Long-term Lyapunov intercepts *ι*_*lt*_ also show an increasing trend across relative power intervals.

**Fig 5:**
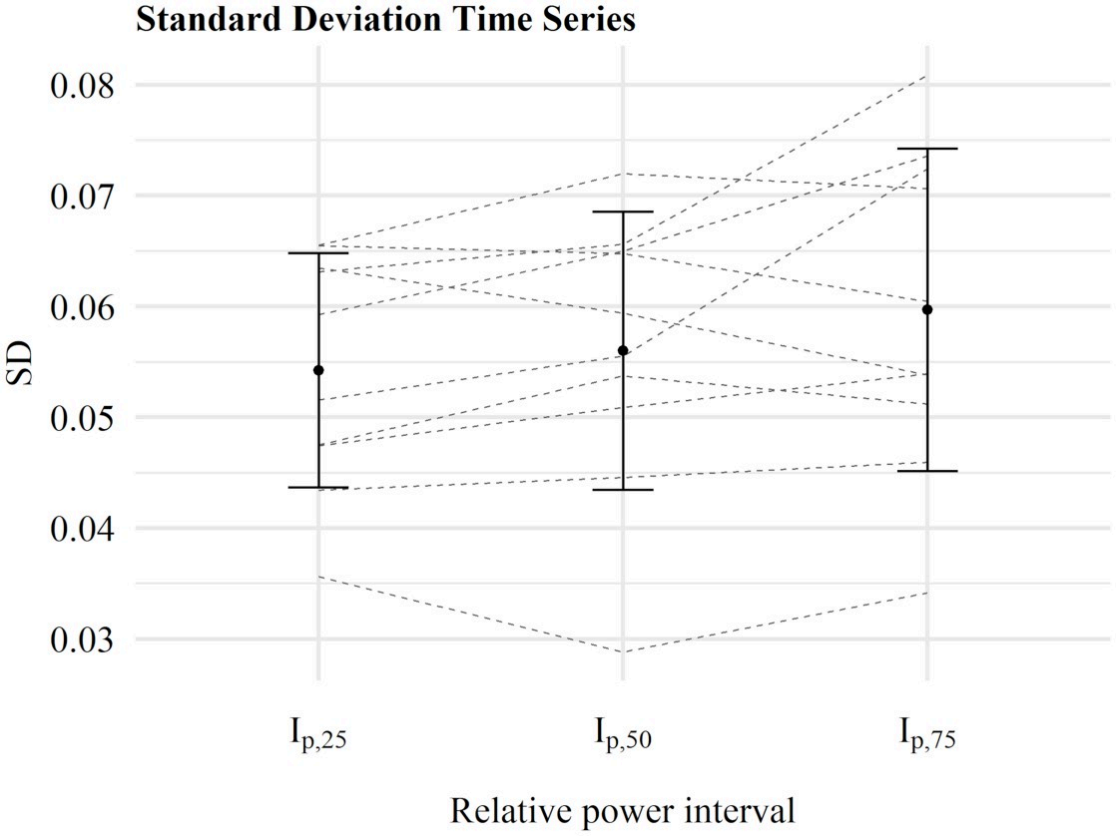
Results of the calculation of the standard deviation. An increase of the standard deviation across relative power intervals was present for 5 out of 10 participants.

Arithmetic mean and standard deviations across three relative power intervals (*I*_*p*,25_, *I*_*p*,50_, *I*_*p*,75_) of respective nonlinear parameters are shown in Figs 3–5. Single participants are displayed as dashed lines. All necessary requirements of the statistical tests were met. Detailed results of the contrast analysis are also presented in Table 3.

**Table 3:** Results of the contrast analysis.

Parameter	t-value	p-value	g-effect
*ML*1_*F*_	4.489	8 × 10^−4^	1.420
*ML*1_*α*_	2.571	0.015	0.813
λ_*st*_	2.196	0.028	0.695
λ_*lt*_	0.206	0.421	0.065
*ι*_*st*_	7.359	2 × 10^−5^	2.327
*ι*_*lt*_	5.834	1 × 10^−4^	1.845
*SD*	1.758	0.056	0.556

### ML1

The results of *ML*1_*F*_ are shown in Fig 3A. A linear increase was found for mean *ML*1_*F*_ across the relative power intervals for all but one participant. Examining all participants, an interindividual variability across the relative power intervals was present, but remained fairly stable with a standard deviation of 0.042, 0.048, and 0.060. Further, the contrast analysis confirmed the presumed linear increase of *ML*1_*F*_ across the steps of the three *I*_*p*,*rel*_ (p-value = 8 × 10^−4^, t-value = 4.489) with a large effect (g-effect = 1.420). The same tendency was observed for *ML*1_*α*_ (Fig 3B). However, the standard deviation increased with increasing load level. The contrast analysis also confirmed a statistically significant linear increase between *ML*1_*α*_ and relative power intervals with p = 0.0151 (t-value = 2.571 and a large effect g = 0.813).

### Lyapunov parameter

The four Lyapunov parameters λ_*st*_, λ_*lt*_, *ι*_*st*_ and *ι*_*lt*_ across the individual power interval are shown in Fig 4. No linear increase was found for the divergence parameter λ_*lt*_ (Fig 4B). In addition, the contrast analysis did not show significant linear differences between the relative power intervals (*p*_λ,*lt*_ = 0.421). The effect and test sizes were small (*g*_λ,*lt*_ = 0.065 and *t*_λ,*lt*_ = 0.206).

For λ_*st*_ (see Fig 4A), however, a linear trend is present ($p_{\lambda_{st}}$ = 0.028, *t*_λ,*st*_ = 2.196 and *g*_λ,*st*_ = 0.695). A distinct linear trend is visible for both intercepts *ι*_*st*_ and *ι*_*lt*_ in Fig 4C and 4D. This trend is also statistically significant with a higher effect *g* for *ι*_*st*_ (*p*_*ι*,*st*_ = 2 × 10^−5^ with *g*_*ι*,*st*_ = 2.327 and *p*_*ι*,*lt*_ = 1 × 10^−4^ with *g*_*ι*,*lt*_ = 1.845).

### Standard deviation

As shown in Fig 5, there was no clear increase of the standard deviation across relative power intervals. Even though 5 out of 10 participants showed an increasing trend across all three intervals, this was not confirmed for the other 5 participants. Consequently, the contrast analysis for a linear relationship between the relative power intervals and the standard deviation shows no significant result (p = 0.0563).

## Discussion

The present paper aimed to answer two main research questions: 1. To what extent can the underlying force data of the previously established *ML*1_*F*_ be substituted by kinematic data to calculate *ML*1_*α*_ and still distinguish individual relative power intervals in cycling? 2. Can other variables, such as Lyapunov parameters, determined via kinematic data also distinguish individual relative power intervals? To answer these questions, an acceleration time series was calculated from the output of a crank-based gyroscope in a cycling laboratory step test. Previous results from laboratory and field studies were confirmed: a statistically significant, linear relationship between *ML*1_*F*_ and the relative power intervals was found although the effects in the current step test were slightly smaller. This indicates a very good reproducibility of *ML*1_*F*_ even under different experimental conditions. Additionally, a linear increase for *ML*1_*α*_ using acceleration data generated from the gyroscope was shown across the three increasing individual load levels. This shows the robustness of *ML*1 towards various signals, as *ML*1_*α*_ seems able to distinguish load levels. The use of a simpler gyroscope or accelerometer on the crank appears justified.

Furthermore, statistically significant linear increase for the short-term Lyapunov exponent λ_*st*_, as well as for short and long-term intercepts of divergences *ι*_*st*_ and *ι*_*lt*_ were found for increasing individual load levels *I*_*p*,25_, *I*_*p*,50_ and *I*_*p*,75_. The same results were not found for the long-term Lyapunov exponent λ_*lt*_. This supports the idea that increasing load during cycling can be described using Lyapunov exponents calculated from kinematic data.

Nevertheless, there are some limiting factors that should be mentioned. λ_*st*_ is calculated as a possible surrogate for local system stability, while λ_*lt*_ is more related to the complexity of the attractor of the corresponding movement [21]. Transferred to the present study, decreasing local motion stability would have to be assumed with increasing load during cycling. However, the complexity of the attractor in phase space does not increase. At the same time, the intercepts *ι*_*st*_ and *ι*_*lt*_ show a significant linear increase with increasing load. While the slope (λ) is a measure of change of divergence in phase space, the intercept *ι* rather describes the ‘initial distance’ or offset of diverging points in this phase space. This could be important within the present calculation of the Lyapunov parameters using the implementation of [22], since the radius for where to find the nearest neighbors (eps) has to be defined. In the presented algorithm, this radius was set to half the standard deviation of the corresponding time series. As shown in Fig 5, half the participants show an increase over the relative power intervals. Therefore, the following applies for these participants: The larger the standard deviation, the larger the radius in which the nearest neighbors can be found. However, with a fixed assumed eps (half mean of overall *SD*) for all conditions, the linear tendency of the intercepts still occurs. Thus, this does not explain the linear increase of *ι*.

Another point to mention is that methodological differences in calculating Lyapunov exponents lead to a lack of comparability [21, 30]. The methodology used in this analysis was transferred from gait analysis and applied to crank acceleration data during cycling. Even though our initial hypothesis was confirmed, the methodology should be adapted and standardized. In this way, different conditions, such as field vs. laboratory settings, may be more comparable.

**Fig 6:**
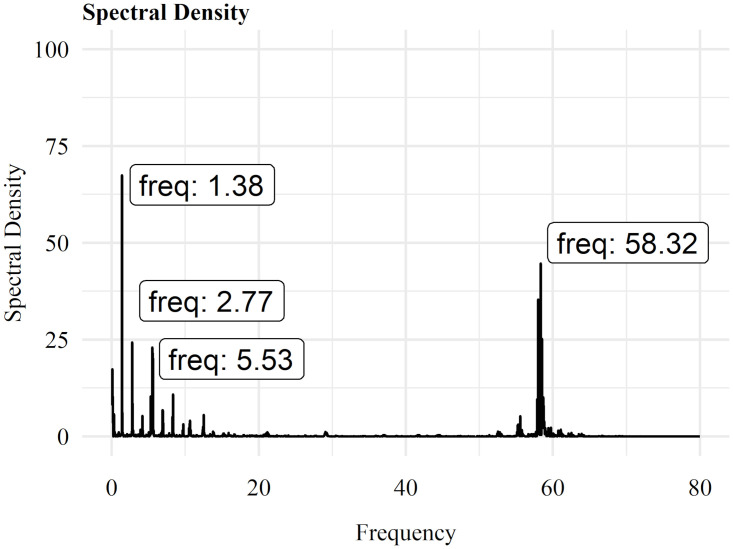
Spectral analysis. Exemplary time series with labeled characteristic frequencies.

Moreover, the pedaling motion is highly constrained, in particular when using an ergometer, as the crank is (nearly) exclusively accelerated in the sagittal plane. Without any axial inclination and a constant crank length in the static position on the ergometer, the motion is restricted to a static circle. Nevertheless, oscillations and vibrations may even occur for a static ergometer, and may have an impact on the results. To exclude such artefacts, a frequency analysis with a Fast Fourier Transformation was performed for each time series of the relative power intervals. No indications for unexplainable peaks could be found. Since the participants executed a cadence between 60 and 90 cycles per minute, a frequency peak can be distinguished at approximately 1 to 1.3 Hz (see Fig 6). Using a 42 teeth chainring, another frequency peak at about 50 to 55 Hz can be explained by multiplying the number of teeth by the cadence. No other unexplainable characteristic frequencies were found. The mentioned frequencies were eliminated using the low-pass filter.

The high number of restrictions in the current laboratory study limits the transfer of our results to field applications. In the field, various confounding environmental variables, such as wind, uneven surfaces, changes in direction, and acceleration, etc., can affect the kinematic signal, in particular through different vibrations. When calculating Lyapunov exponents, these influences add to the complexity of the system. It is impossible to say whether and in what manner these conditions affect the results of the Lyapunov exponents. Acceleration signals and *ML*1_*α*_ may be more exposed to external factors than force data and *ML*1_*F*_. This may also be the reason for the clearer linear increase of *ML*1_*F*_ compared to *ML*1_*α*_ in the results even in the current laboratory test. This is also suggested in [11], where a replication of *ML*1_*F*_ within indoor and outdoor tests was shown—despite external disturbance factors and additional vibrations. However, the disadvantage of *ML*1_*α*_ in field applications remains hypothetical at this point and should be further investigated in field studies.

With respect to future applications of the present findings, further consideration should be given to the extent to which they can be used to optimize the algorithms of e-bike propulsion. Currently, every available time series was considered in its entirety to maintain the system’s stability. These 3-minutes intervals contained approximately 255 cycles, i.e. 255 revolutions of the crank, and were thus too long for a real-time application. A sensitivity analysis should be performed to examine the required length of the time series that is needed to still distinguish relative power intervals in the same way as when considering the entire time series. The numbers of cycles should be determined from the middle of the corresponding relative power interval, in order to avoid possible short-term adjustments to load changes. According to a first assessment, a length of about 75 to 100 cycles seems to be necessary. This corresponds to a slightly longer time series than the length proposed in [11] for *ML*1_*F*_. Nevertheless, further research is needed. This also applies to the generalizability of our findings, since our sample was fairly small and represented a rather homogeneous group of participants (males only).

## Conclusions

To efficiently optimize e-bike algorithms, it is beneficial to determine the individual load of the cyclist using direct mechanical bicycle data, so there is no need for additional devices. Our study found that nonlinear dynamical methods are suitable for detecting individual load in cycling using mechanical kinematic data. We observed a linear increase in *ML*1, the short term Lyapunov Exponent λ_*st*_ and slopes (*ι*_*st*_ and *ι*_*lt*_) across three relative power intervals based on data from a laboratory ergometer step test.

For both parameters, intraindividual differences were shown across three relative power intervals, but with notable interindividual differences of the participants within the same relative power interval. Thus, there is a noticeable trend for individual participants across increasing load levels, but with different respective base levels. However, the interindividual difference is not related to the individually achieved absolute power output of each participant in the ergometer step test. The nonlinear methods used in this context may accurately describe the individual load.

However, there are limitations to the transferability of these findings, as the data from the gyroscope used in this study were collected in a laboratory setting. In field conditions, or simply when not cycling on an ergometer, there may be additional influences (such as stronger vibrations), that affect the system stability. The high variance of *ML*1_*α*_ at the highest relative power interval (*I*_*p*,75_) may be an indication of the presence of increasing interactions of hidden influences, even since no unexplained frequencies were found in the FFT. Further field tests are needed to determine the extent to which such influences may affect the system stability described by λ_*st*_.

In summary, results of this paper show that λ_*st*_ in addition to the *ML*1_*F*_ may be useful to optimize of e-bike algorithms with respect to individual load based on kinematic data. However, further field tests and analysrs of real-time capability are needed to validate these findings.

## Supporting information

S1 Fig Absolute power output over relative power intervals. The absolute mean values of the power outputs in *W*/*kg* increase equally over the relative power intervals and the variances remain constant.
(TIFF)

S2 Fig Discriminating statistics of the surrogate and original data. For each participant, the statistic value of the original data is outside the values of the surrogate data.
(TIFF)

S1 Table Absolute power outputs across relative power intervals in *W* and *W*/*kg*. (PDF)
