# Supplementary figures and images for: Quantifying workload using nonlinear dynamical measures of biomechanical parameters during cycling on a roller trainer
Source: PLoS One. 2023 May 9;18(5):e0285408. doi: 10.1371/journal.pone.0285408 (PMC10168574; doi:10.1371/journal.pone.0285408)

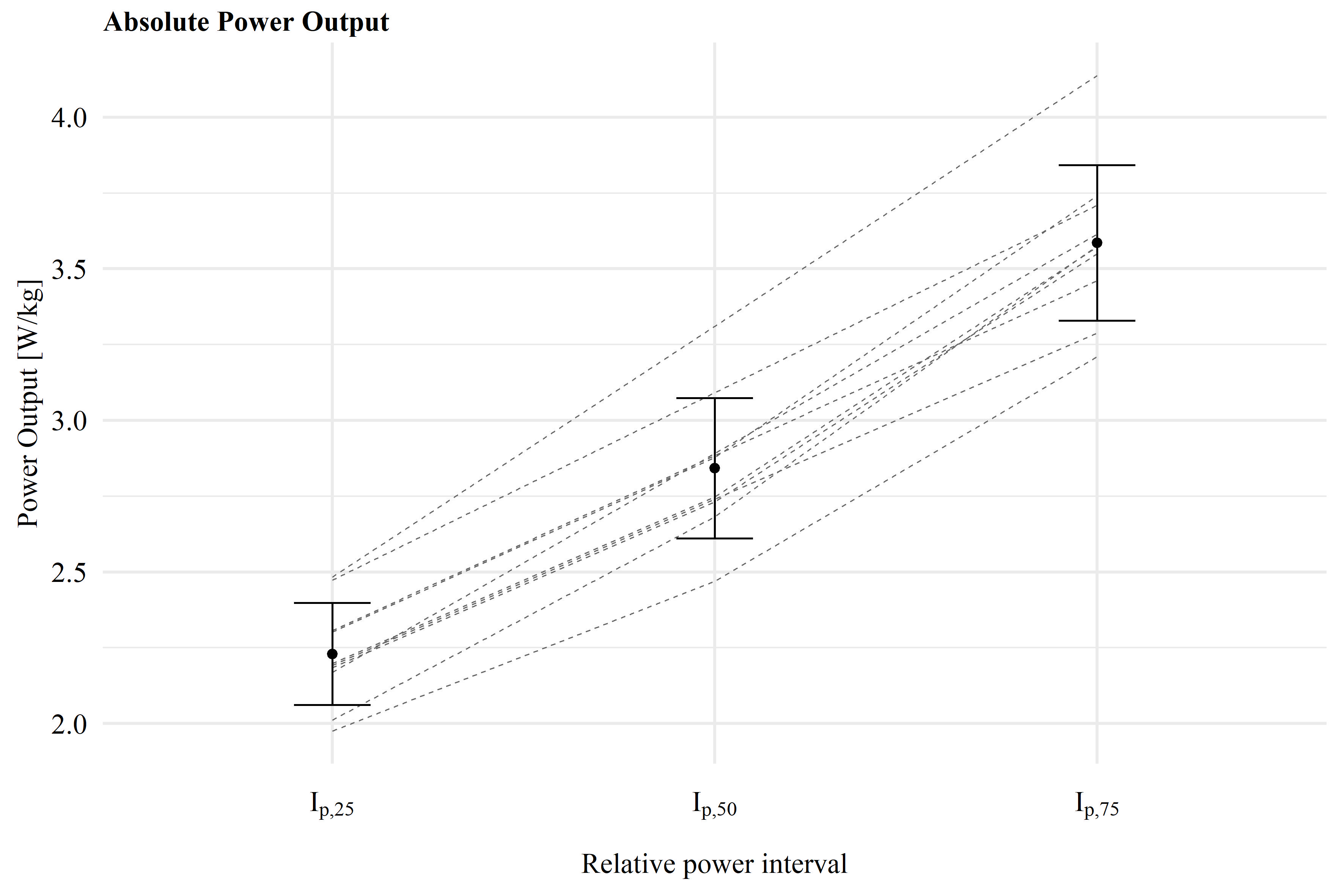

Supplement: S1 Fig — The absolute mean values of the power outputs in W/kg increase equally over the relative power intervals and the variances remain constant. (TIFF) [file pone.0285408.s001.tiff]

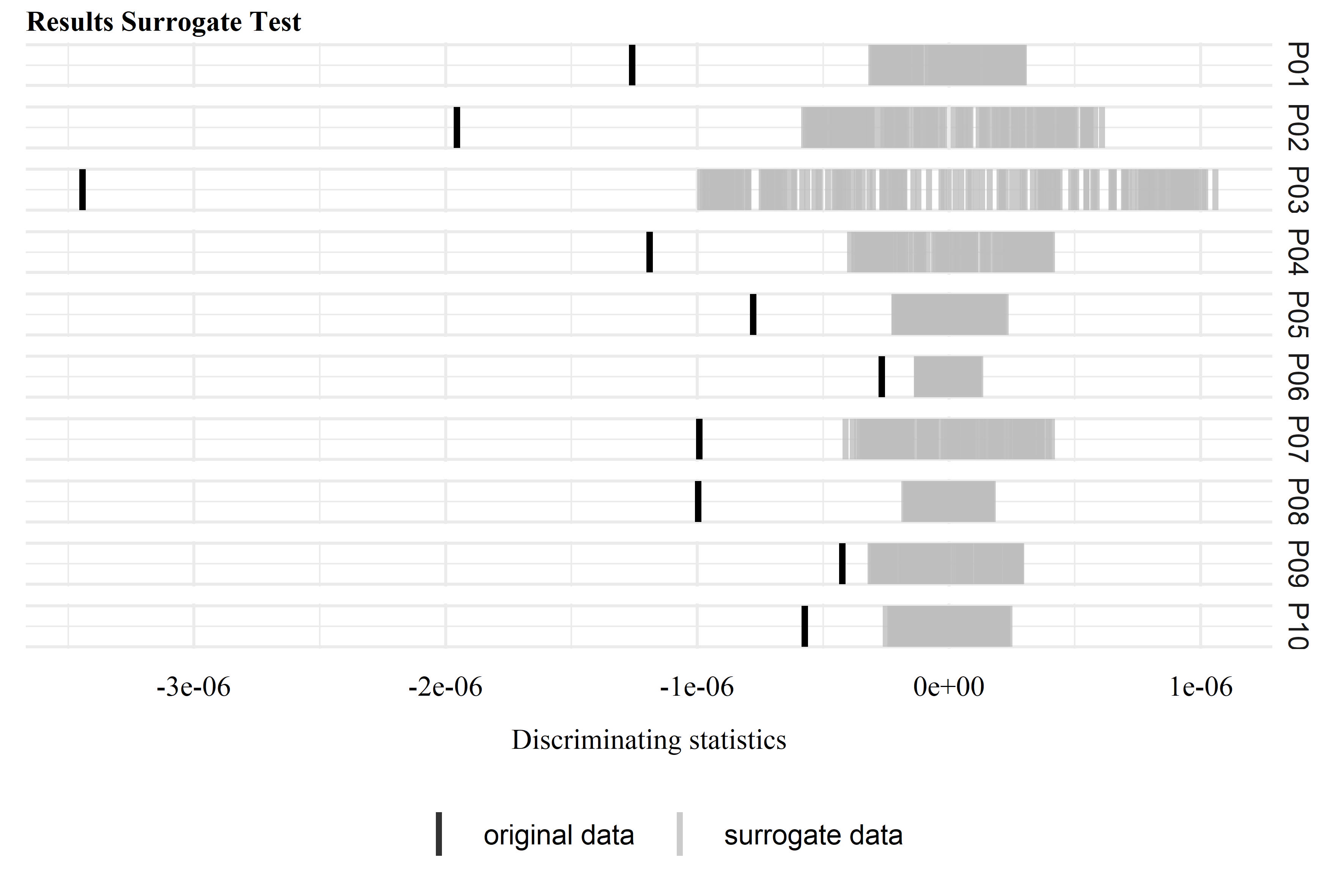

Supplement: S2 Fig — For each participant, the statistic value of the original data is outside the values of the surrogate data. (TIFF) [file pone.0285408.s002.tiff]
