# Supplementary material for: Quantifying workload using nonlinear dynamical measures of biomechanical parameters during cycling on a roller trainer
Source: PLoS One. 2023 May 9;18(5):e0285408. doi: 10.1371/journal.pone.0285408 (PMC10168574; doi:10.1371/journal.pone.0285408)
Supplement: S1 Table — (PDF) [file pone.0285408.s003.pdf]

S2 Table. Absolute power outputs across relative power intervals in W and W/kg.

P	$I_{p,25}[W]$	$I_{p,50}[W]$	$I_{p,75}[W]$	$I_{p,25}[W/kg]$	$I_{p,50}[W/kg]$	$I_{p,75}[W/kg]$
$P01$	160	200	240	2.47	3.09	3.71
$P02$	160	200	260	2.20	2.75	3.57
$P03$	180	240	300	2.17	2.89	3.61
$P04$	160	200	260	2.18	2.73	3.55
$P05$	180	240	320	2.01	2.68	3.58
$P06$	160	200	240	2.19	2.74	3.29
$P07$	160	200	240	2.31	2.88	3.46
$P08$	160	200	260	1.98	2.47	3.21
$P09$	180	240	300	2.48	3.31	4.14
$P10$	180	200	260	2.30	2.88	3.74
